# Identification of a cyclic-di-GMP-modulating response regulator that impacts biofilm formation in a model sulfate reducing bacterium

**DOI:** 10.3389/fmicb.2014.00382

**Published:** 2014-07-29

**Authors:** Lara Rajeev, Eric G. Luning, Sara Altenburg, Grant M. Zane, Edward E. K. Baidoo, Michela Catena, Jay D. Keasling, Judy D. Wall, Matthew W. Fields, Aindrila Mukhopadhyay

**Affiliations:** ^1^Physical Biosciences Division, Lawrence Berkeley National LaboratoryBerkeley, CA, USA; ^2^Center for Biofilm Engineering, Montana State UniversityBozeman, MT, USA; ^3^Department of Biochemistry, University of MissouriColumbia, MO, USA; ^4^Department of Chemical and Biomolecular Engineering, Department of Bioengineering, University of CaliforniaBerkeley, CA, USA; ^5^Department of Microbiology and Immunology, Montana State UniversityBozeman, MT, USA

**Keywords:** cyclic-di-GMP, biofilm, *Desulfovibrio*, HD-GYP, GGDEF, GGDEF-EAL, response regulator, two-component system

## Abstract

We surveyed the eight putative cyclic-di-GMP-modulating response regulators (RRs) in *Desulfovibrio vulgaris* Hildenborough that are predicted to function via two-component signaling. Using purified proteins, we examined cyclic-di-GMP (c-di-GMP) production or turnover *in vitro* of all eight proteins. The two RRs containing only GGDEF domains (DVU2067, DVU0636) demonstrated c-di-GMP production activity *in vitro*. Of the remaining proteins, three RRs with HD-GYP domains (DVU0722, DVUA0086, and DVU2933) were confirmed to be Mn^2+^-dependent phosphodiesterases (PDEs) *in vitro* and converted c-di-GMP to its linear form, pGpG. DVU0408, containing both c-di-GMP production (GGDEF) and degradation domains (EAL), showed c-di-GMP turnover activity *in vitro* also with production of pGpG. No c-di-GMP related activity could be assigned to the RR DVU0330, containing a metal-dependent phosphohydrolase HD-OD domain, or to the HD-GYP domain RR, DVU1181. Studies included examining the impact of overexpressed cyclic-di-GMP-modulating RRs in the heterologous host *E. coli* and led to the identification of one RR, DVU0636, with increased cellulose production. Evaluation of a transposon mutant in DVU0636 indicated that the strain was impaired in biofilm formation and demonstrated an altered carbohydrate:protein ratio relative to the *D. vulgaris* wild type biofilms. However, grown in liquid lactate/sulfate medium, the DVU0636 transposon mutant showed no growth impairment relative to the wild-type strain. Among the eight candidates, only the transposon disruption mutant in the DVU2067 RR presented a growth defect in liquid culture. Our results indicate that, of the two diguanylate cyclases (DGCs) that function as part of two-component signaling, DVU0636 plays an important role in biofilm formation while the function of DVU2067 has pertinence in planktonic growth.

## Introduction

**Figure 1:**
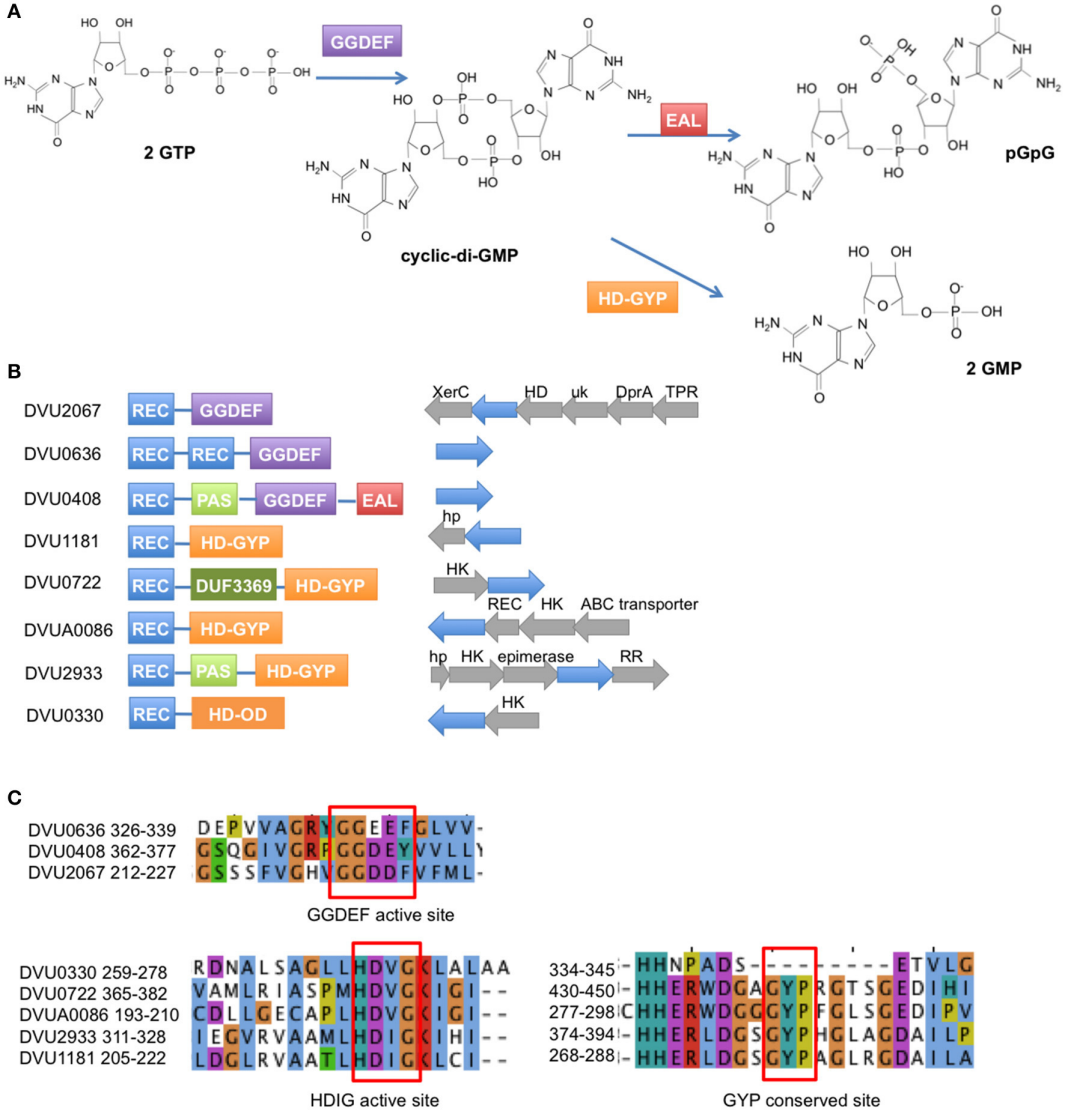
**Response regulators in *D. vulgaris* Hildenborough with GGDEF, EAL, and HD domains. (A)** The GGDEF domain synthesizes c-di-GMP from GTP, while the EAL and HD-GYP domains hydrolyze c-di-GMP to pGpG and GMP, respectively. **(B)** Domain structures are shown for the 2 GGDEF domain RRs, 1 GGDEF-EAL RR, 4 HD-GYP RRs, and 1 HDOD RR (REC = receiver domain). To the right of the domain structures are shown the operon arrangements of each RR with the RR gene shown in blue. (Abbreviations: XerC, integrase domain; HD, HD domain; uk, unknown domain; DprA, DNA processing protein A; TPR, Tetratricopeptide domain protein; hp, hypothetical protein; HK, histidine kinase; REC, receiver domain). **(C)** Amino acid sequence alignment of the conserved active site residues for the GGDEF domain RRs (top) and the HD domain RRs (bottom). Note that DVU0408 has a Y instead of the conserved F in its GGDEF domain. Also DVU0330 is an HDOD domain that lacks the GYP motif.

Many inter- and intra- species interactions are mediated by small molecule signals. Of these, cyclic-di-GMP (c-di-GMP) has come to be recognized as a ubiquitous second messenger in bacteria. Discovered in 1987 in *Gluconacetobacter xylinus*, the first established function of c-di-GMP was in the allosteric activation of a cellulose synthase for the biosynthesis of extracellular cellulose (Ross et al., 1987). C-di-GMP is now known to play a role in a large number of phenotypes ranging from attachment, motility, biofilm formation, quorum sensing to numerous other developmental, pathogenic, and virulence responses (for a recent review see Romling et al., 2013). In general, accumulation of c-di-GMP results in greater sessility, attachment and improved biofilm formation, while reduction of the same results in greater motility and other associated growth characteristics (Simm et al., 2004). Not surprisingly, enzymes that modulate c-di-GMP levels have been annotated in most bacteria (Galperin et al., 2010) and have been well reviewed (Hengge, 2009; Schirmer and Jenal, 2009). Levels of c-di-GMP in the cell are modulated through the function of GGDEF motif-containing proteins that act as diguanylate cyclases (DGCs) with GTP as substrate (Paul et al., 2004; Ryjenkov et al., 2005), and EAL (Ross et al., 1987; Simm et al., 2004; Schmidt et al., 2005) or HD-GYP motif-containing proteins (Ryan et al., 2006; Stelitano et al., 2013) that act as phosphodiesterases (PDEs) that degrade c-di-GMP to pGpG or GMP (Figure 1A).

C-di-GMP modulating domains are most commonly found in combination with other signaling domains, such as PAS or GAF, or as output domains in the receiver portion of two-component system response regulators (RRs) (Romling et al., 2013). Signaling systems that use second messenger signaling are critical to survival in different environments and provide a competitive advantage to biological systems under different environmental constraints. However, few such sets of enzymes have been functionally explored (Solano et al., 2009; Wang et al., 2010; Tan et al., 2014).

Here we examine all presumptive c-di-GMP-modulating RRs that are part of two-component signaling in the sulfate-reducing bacterium *Desulfovibrio vulgaris* Hildenborough. Sequenced in 2003 (Heidelberg et al., 2004), this bacterium serves as a model organism to study sulfate reduction and carbon oxidation as well as metal reduction (Beyenal et al., 2004), metal corrosion (Lee et al., 1995), biofilm formation (Clark et al., 2012), and bioimmobilization in superfund sites (Faybishenko et al., 2008). *D. vulgaris* Hildenborough has been extensively studied for a variety of stress responses (Zhou et al., 2011), syntrophic interactions (Hillesland and Stahl, 2010), regulatory motifs (Rodionov et al., 2004; Kazakov et al., 2013b), and signal transduction (Rajeev et al., 2011). The genome of *D. vulgaris* Hildenborough has a high “microbial sensory IQ” (Galperin et al., 2010) encoding a total of 41 genes containing GGDEF/EAL/HD-GYP domains. Of these, eight are linked to two-component system response regulator receiver domains (Figure 1B) and none have been functionally characterized. We used purified proteins to evaluate the predicted function of all eight candidates. We also conducted *in vivo* assays to test certain phenotypes that are known to be associated with c-di-GMP modulation. We identified DVU0636 to be involved in biofilm formation in *D. vulgaris* Hildenborough.

## Materials and methods

### Cloning of response regulator genes

Full-length genes for the eight RRs were cloned by Gateway technology into the destination vector pETDEST42 (Life Technologies, Grand Island, NY, USA) as previously described (Rajeev et al., 2011), such that the protein is expressed with a C-terminal V5 epitope and 6X-His tag. The HD-GYP domains of DVUA0086 (164–363 aa) and DVU2933 (282–458 aa) were cloned into pSKB3 (Kan^R^) with an N-terminal cleavable 6X-His tag. The HD-GYP domains of DVU1181 (176–356 aa), DVU0722 (336–518 aa) and DVU0330 (230–412) were cloned into pSKB3 with a cleavable N-terminal 8x-His-tag, Strep Tag II, and a maltose binding protein tag. The genes were amplified from *D. vulgaris* Hildenborough genomic DNA with iProof HiFidelity polymerase (BioRad, Hercules, CA, USA) and primers that were designed to have 40 bp overlap between the insert and the vector backbone. The vector was similarly amplified and treated with DpnI, followed by agarose gel extraction and purification. The vector and insert were mixed in a 1:1 molar ratio and assembled with a modified Gibson reagent (Gibson et al., 2009) that lacked the Taq ligase. The Gibson reaction was used to transform *E. coli* BL21 (DE3) electrocompetent cells and transformants were selected on Yeast Tryptone agar plates with kanamycin (50 μg/ml). The presence of the insert was verified by sequencing. Note on DVU2067 cloning: Sequencing of the cloned DVU2067 revealed an extra 39 bp sequence at the C-terminus that appeared to be a repetition of the last 19–20 bp of the gene.

### Protein overexpression

Expression constructs were grown in 10 ml Terrific Broth (TB) (Tartof and Hobbs, 1987) containing kanamycin (50 μg/ml) at 37°C until an OD_600_ of 1.5. Protein expression was induced with 0.25 mM IPTG, and cells were grown at 22°C overnight. The cells were harvested, resuspended in 1× PBS buffer and lysed by sonication. The lysates were centrifuged at 15,000 × *g* for 30 min at 4°C. The clarified lysate was examined for protein overexpression by SDS-PAGE followed by Western blotting with mouse anti-His monoclonal antibodies.

### Protein purification

The expression strain was grown in 1 L of TB with kanamycin (50 μg/ml) at 37°C until OD_600_ of 1.5. IPTG (0.25 mM) was added and cells were grown at 22°C overnight. Cells were harvested and lysed by sonication in 150 ml of cold Buffer A (20 mM sodium phosphate, 0.5 M NaCl, 40 mM imidazole, pH 7.4) with lysozyme (1 mg/ml) and 1× benzonase nuclease (New England Biolabs, Ipswich, MA, USA). Lysate was clarified by centrifugation at 15,000 × *g* for 30 min at 4°C and filtered on a 0.45-μm pore-sized syringe filter. The lysate was loaded onto a 5 ml HisTrapFF column (GE Life Sciences, Piscataway, NJ) in an Akta Explorer 100 FPLC instrument (GE Life Sciences, Piscataway, NJ) and eluted with a gradient of 0–100% Buffer B (20 mM sodium phosphate, 0.5 M NaCl, 500 mM imidazole, pH 7.4) over 30 min with a flow rate of 2 ml/min. Purified fractions were pooled and buffer exchanged on a 26/10 desalting column (GE Life Sciences) into a desalting buffer (20 mM sodium phosphate pH 7.4, 300 mM NaCl). The tags were removed by incubating overnight with purified His-tagged TEV protease at 4°C with rocking. The TEV protease, cleaved tags, and uncleaved proteins were removed by passing the mixture through a 5 ml HisTrapFF column. Tagless proteins were buffer exchanged on a 26/10 desalting column into 20 mM Tris-HCl pH 8.0 and 20 mM NaCl. Proteins were concentrated to ~0.5–1 mg/ml, glycerol was added to 10% (vol/vol), aliquots were flash frozen in liquid nitrogen and stored at −80°C.

For the HD-GYP domains of DVU1181, DVU0722, and DVU0330, a second round of affinity purification was performed by eluting from the first HisTrapFF column onto a 5 ml StrepTrapFF (GE Life Sciences). The column was washed with Buffer C (20 mM sodium phosphate pH 7.4, 280 mM NaCl, 6 mM KCl) and eluted with Buffer D (Buffer C + 2.5 mM desthiobiotin). Then, 50 mM L-arginine and 50 mM L-glutamic acid buffered in Tris-HCl were added to stabilize proteins (Golovanov et al., 2004). This was followed by TEV cleavage and purification of tagless protein as described above.

### DGC assay for DVU0636, DVU2067, and DVU0408

Full length DVU0636, DVU2067, and DVU0408 with a C-terminal V5 epitope and 6x His tag were purified from *E. coli*. Purified protein (1.6–1.8 μM) was mixed with 0.5 mM GTP in 50 mM Tris HCl pH 8.0, 100 mM NaCl, and either 2 mM MgCl_2_ or 2 mM MnCl_2_ in a total volume of 100 μl. The reactions were incubated at 30°C for 24 h. The proteins were denatured by heating at 95°C for 5 min. The samples were centrifuged at 15,000 × *g* for 10 min, and the supernatant was filtered through a 10 K molecular weight cutoff centrifugal filter prior to HPLC analysis. Samples (3 μl) were injected into an Inertsil ODS-3 column (3 μ, 250 × 2.1 mm; GL Sciences, Torrance, CA) equipped with a guard column (Inertsil ODS-3, 3 μ, 50 × 2.1 mm; GL Sciences) on an HPLC system (Agilent Technologies, Santa Clara, CA). Buffer A: 100 mM Potassium phosphate buffer pH 6.0; Buffer B = 100% (vol/vol) methanol. The samples were run for 25 min at a flow rate of 0.2 ml/min, with the following gradient: 0 min – 2% (vol/vol) B; 2 min – 2% B; 14 min – 30% B; 17 min – 30% B; 18 min – 2% B; 25 min – 2%B.

### PDE activity assay: with bis-*p*NPP

Triplicate enzyme reactions for each protein were set up in a 96-well microplate (Costar, black with clear flat bottom, polystyrene; Corning Incorporated, Corning, NY). Proteins (7–10 μg) were mixed in a total volume of 100 μl with 5 mM bis-*p*NPP in 25 mM Tris-HCl pH 8.0, 100 mM NaCl, 1 mM DTT, and either 1 or 10 mM MnCl_2_ or 10 mM MgCl_2_. Absorbance was measured at 410 nm every 10 min in a SpectraMax Plus plate reader (Molecular Devices, Sunnyvale, CA, USA). A reaction that lacked bis-*p*NPP served as the blank, while a reaction with no protein served as a negative control.

### PDE activity assay: with c-di-GMP

Each protein (7–10 μg) was mixed with 100 μM c-di-GMP in 25 mM Tris HCl pH 8.0, 10 mM MnCl_2_, 100 mM NaCl, 1 mM DTT in a total volume of 100 μl HPLC grade water. Reactions were carried out in triplicate and incubated at room temperature for 24 h. The reactions were stopped and proteins denatured by heating to 95°C for 5 min. The samples were centrifuged at 15,000 × *g* for 10 min, and the supernatant was filtered through a 10 K molecular weight cutoff spin filter before HILIC-TOF MS (hydrophilic interaction liquid chromatography and time of flight mass spectrometry) analysis. The proteins were also tested for activity against 100 μM of cyclic-di-AMP, cyclic-AMP, cyclic-GMP, and pGpG. Samples were injected onto a SeQuant Zic pHILIC (150 mM length × 2.1 mM diameter column together with a 20 × 2.1 mM Zic pHILIC guard column; EMD Millipore, Billerica, MA, USA). For a full description of the analytical method, please refer to Bokinsky et al. (2013).

### Congo red *E. coli* assay

*E. coli* BL21 Star DE3 strains expressing the full-length RR genes were grown overnight in 3 ml LB containing carbenicillin (100 μg/ml). Each strain was diluted 1:100 in fresh medium, grown until OD_600_ of 0.5 at 37°C, and then streaked out on LB-carbenicillin plates containing Congo Red (CR) 5 μg/ml (±0.5 mM IPTG). Plates were incubated at room temperature for 4 days.

### Transposon mutant library

The transposon mutants were obtained from the *D. vulgaris* transposon mutant collection (Zane and Wall, 2013) constructed at the University of Missouri, which have been used in other studies (Fels et al., 2013; Kazakov et al., 2013a; Ray et al., 2014). Strain descriptions are provided in Table 1.

**Table 1:** **Transposon mutant strains used in this study**.

**Name**	**Description**
GZ0620	DVU0330-496::Tn5-RL27; insertion at bp 496/1239; Km^r^
GZ2490	DVU2067-828::Tn5-RL27; insertion at bp 828/1122; Km^r^
GZ3062	DVU0636-506::Tn5-RL27; insertion at bp 506/1314; Km^r^
GZ4281	DVU0408-857::Tn5-RL27; insertion at bp 857/2148; Km^r^
GZ4944	DVU1181-479::Tn5-RL27; insertion at bp 479/1071; Km^r^
GZ5161	DVUA0086-105::Tn5-RL27; insertion at bp 105/1092; Km^r^
GZ5218	DVU0722-279::Tn5-RL27; insertion at bp 279/1557; Km^r^
GZ2493	DVU2933-215::Tn5-RL27; insertion at bp 215/1377; Km^r^

### Growth assays for *D. vulgaris* strains

*D. vulgaris* was grown in a defined medium containing 8 mM MgCl_2_, 20 mM NH_4_Cl, 2.2 mM K_2_PO_4_, 0.6 mM CaCl_2_, 30 mM Tris, 1 ml/liter of Thauer's vitamins (Brandis and Thauer, 1981), 12.5 ml/liter of trace element solution (Rajeev et al., 2012), 640 μl/ liter of resazurin (0.1% wt/vol), and supplemented with 50 mM Na_2_SO_4_ and 60 mM sodium lactate (LS4D medium). The pH of the medium was adjusted to 7.2 with 1 N HCl. Cultures were grown at 30°C in an anaerobic growth chamber (COY Laboratory Products, Grass Lake, MI, USA) in an atmosphere of 85% N_2_, 10% CO_2_, and 5% H_2_. For transposon mutants, the medium was supplemented with the antibiotic G418 (400 μg/ml) (Sigma Aldrich, St. Louis, MO, USA). *D. vulgaris* strains were grown from freezer stocks in LS4D supplemented with 0.1% (wt/vol) yeast extract in 15 ml centrifuge tubes and then transferred to LS4D medium with a 2% (vol/vol) inoculum. 400 μl of cultures were transferred (4 replicates each) into 100-well honeycomb well plates (Growth Curves USA, Piscataway, NJ, USA) and growth measurements were conducted on the Bioscreen C instrument (Growth Curves USA, Piscataway, NJ, USA) at 30°C within the anaerobic chamber.

### Complementation of Tn5::DVU0636

DVU0636 was cloned into the *E. coli-D. vulgaris* shuttle vector pMO9075 (Keller et al., 2014) under a constitutive kanamycin promoter, and with a C-terminal His-tag. Strain GZ3062 (Tn5::DVU0636) was grown overnight from a freezer stock in 10 ml MOYLS4 (+G418). Five milliliter of the overnight culture was used to inoculate 45 ml of fresh MOYLS4 (+G418) and allowed to grow overnight. Electrocompetent cells were prepared as previously described (Zane et al., 2010), and cells (50 μl) were transformed with pMO9075::DVU0636. Transformants were allowed to recover in 1 ml MOYLS4 overnight and then were plated on MOYLS4 containing spectinomycin (100 μg/ml). Colonies were obtained after 4–5 days. Colonies were inoculated into 1 ml MOYLS4 (+G418 and spectinomycin), grown overnight, and transferred to 5 ml MOYLS4 (+G418 and spectinomycin). The resulting culture was used for plasmid preparations and to make freezer stocks. The plasmids were verified by sequencing and the complemented strain was designated as GZ3062(pMO9075::DVU0636).

### Biofilm assays

Frozen stocks of *D. vulgaris* Hildenborough WT (from Montana State University), GZ3062, and the complemented strain were grown to approximately 0.6 OD_600_ in LS4D medium before transfer. The mutant and complement were maintained under appropriate antibiotics for the duration of the study. Once transferred, the cells were again grown to 0.6 OD_600_ before inoculation (approximately 10% vol/vol inoculum) into the biofilm reactor. The culture was allowed to grow for approximately 24 h before constant medium flow was introduced at a dilution rate of 0.10 h^−1^. Biofilm samples were grown in a CDC biofilm reactor (Biosurface Technologies, Bozeman, MT, USA) at 30°C in LS4D under constant sparging (~0.85 kPa/min) with anoxic N_2_ as previously described (Clark et al., 2012). Coupon holders fitted with glass microscope slides (cut in half) were removed at approximately 120 h, and samples were washed with chilled phosphate buffered saline (50 mM phosphate, 0.7% NaCl, pH 7.2) to remove loosely attached cells. The biofilm was then scraped from each slide into sterile tubes and stored at −80°C. Biofilm samples for electron microscopy were treated as previously described (Clark et al., 2007). Glass slide sections were mounted on aluminum stubs, coated with iridium, and viewed on a SUPRA 55VP field emission scanning electron microscope (Carl Zeiss Microscopy, Peabody, MA, USA) at 1.0 kV. To determine carbohydrate and protein levels for the different cultures, hexose sugars were measured by the colorimetric cysteine-sulfuric acid method with glucose as the standard (Chaplin, 1986). Protein was measured with the Qubit® Protein Assay Kit (Life Technologies, Grand Island, NY, USA) and Qubit® Fluorometer (Life Technologies, Grand Island, NY, USA) according to the manufacturer's instructions.

## Results

### Biochemical analysis of the c-di-GMP modulating RRs

In order to confirm the putative functions of the annotated RRs with c-di-GMP modulating domains, genes encoding the eight RRs were cloned into *E. coli*, the encoded proteins produced and purified.

#### DVU0636 and DVU2067 have DGC activity, DVU0408 has PDE activity

**Figure 2:**
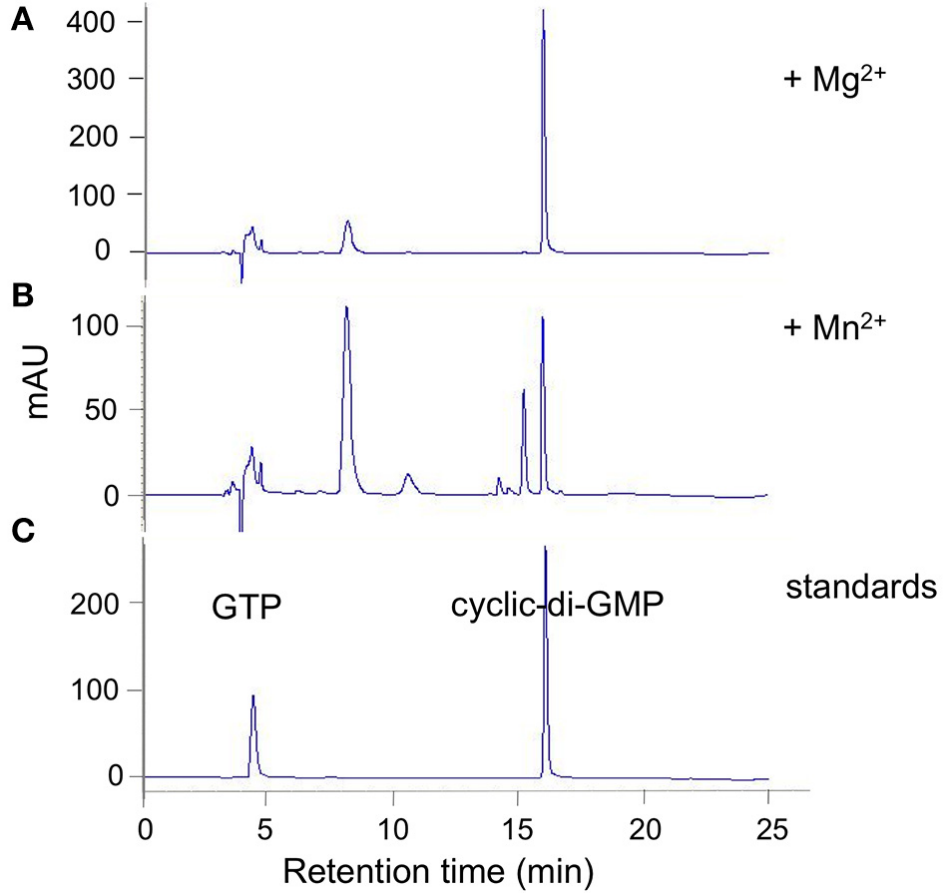
**DVU0636 has DGC activity**. HPLC traces for DGC activity assays with purified DVU0636 (with C-terminal V5 epitope and 6× His tags) and 500 μm GTP in the presence of 2 mM MgCl_2_ **(A)**, or 2 mM MnCl_2_ **(B)**. 100 μm GTP and 100 μm c-di-GMP were used as standards **(C)**.

**Figure 3:**
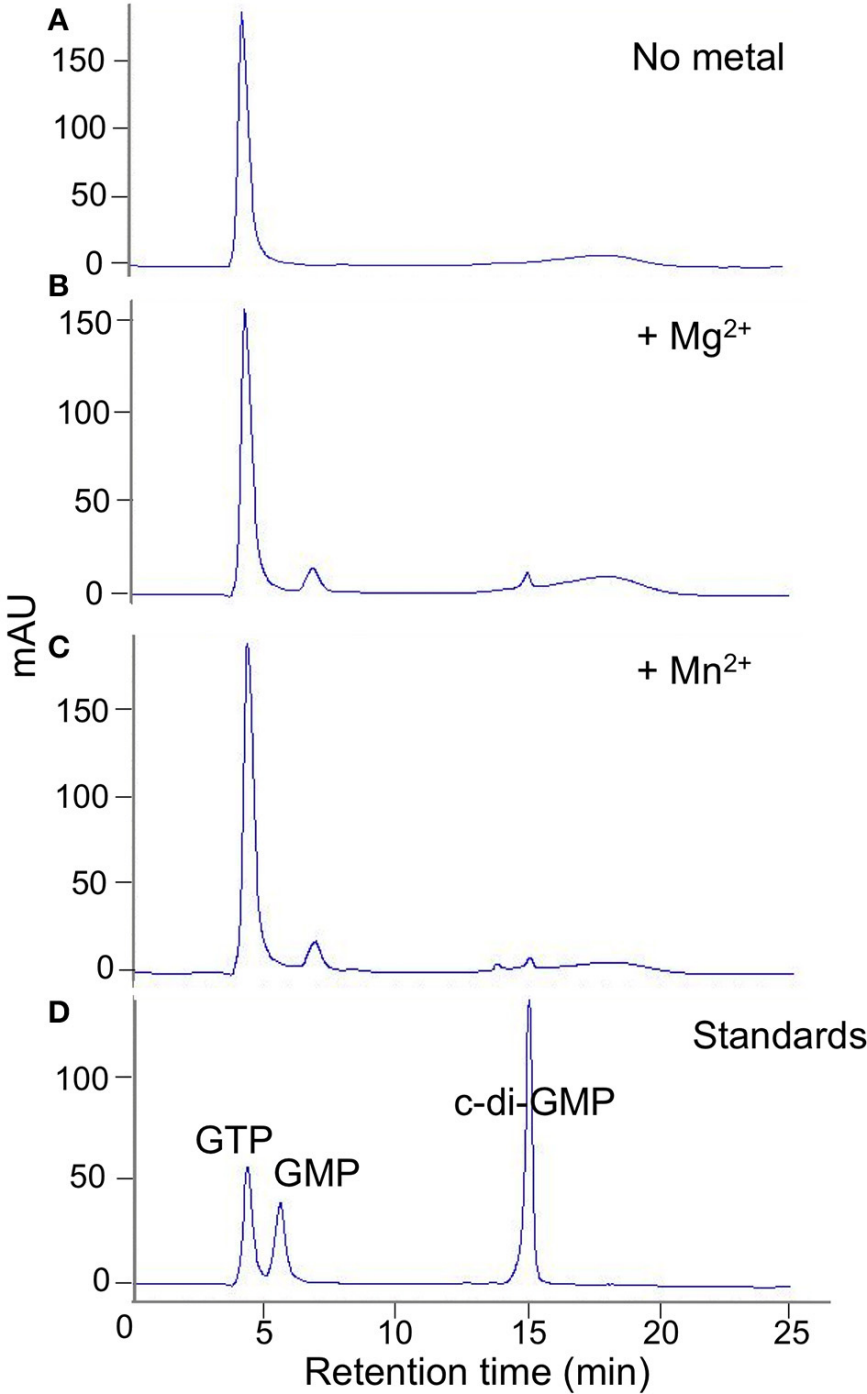
**DVU2067 has very low DGC activity**. HPLC traces for diguanylate cyclase assay with purified DVU2067. Purified DVU2067 (with C-terminal V5 epitope and 6X-His tags) was mixed with 500 μm GTP in the presence of **(A)** no metal; **(B)** 2 mM MgCl_2_; **(C)** 2 mM MnCl_2_; and incubated for 40 h. A mixture of 100 μm GTP, 100 μm GMP, and 100 μm c-di-GMP were run as standards **(D)**.

The three RRs with GGDEF domains were subjected to DGC activity assays by incubation with GTP followed by HPLC analysis (Figures 2, 3).

Purified tagged full length DVU0636 protein converted nearly all of the GTP into c-di-GMP within 24 h of incubation with Mg^+2^ ions (2 mM) (Figure 2A). Interestingly when incubated with Mn^+2^ ions (2 mM), although the GTP was completely consumed, other peaks were present in addition to that of c-di-GMP (Figure 2B). We suspect that these are secondary products rather than reaction intermediates since incubation of the reaction with Mg^+2^ for a shorter time (4 h) did not produce these intermediate peaks (except for a small peak at 8 min), and extending the incubation time with Mn^+2^ to 40 h still resulted in the alternate product peaks (not shown). No product formed in the absence of either Mg^+2^ or Mn^+2^.

When full length purified tagged DVU2067 protein was incubated with GTP, there was very little product formed with either Mn^+2^ or Mg^+2^ ions. The small peak seen appears to be c-di-GMP as it was absent when no metal ions were added to the reaction (Figure 3). The addition of acetyl phosphate to stimulate phosphorylation did not increase the activity of either DVU0636 or DVU2067 (not shown).

**Figure 4:**
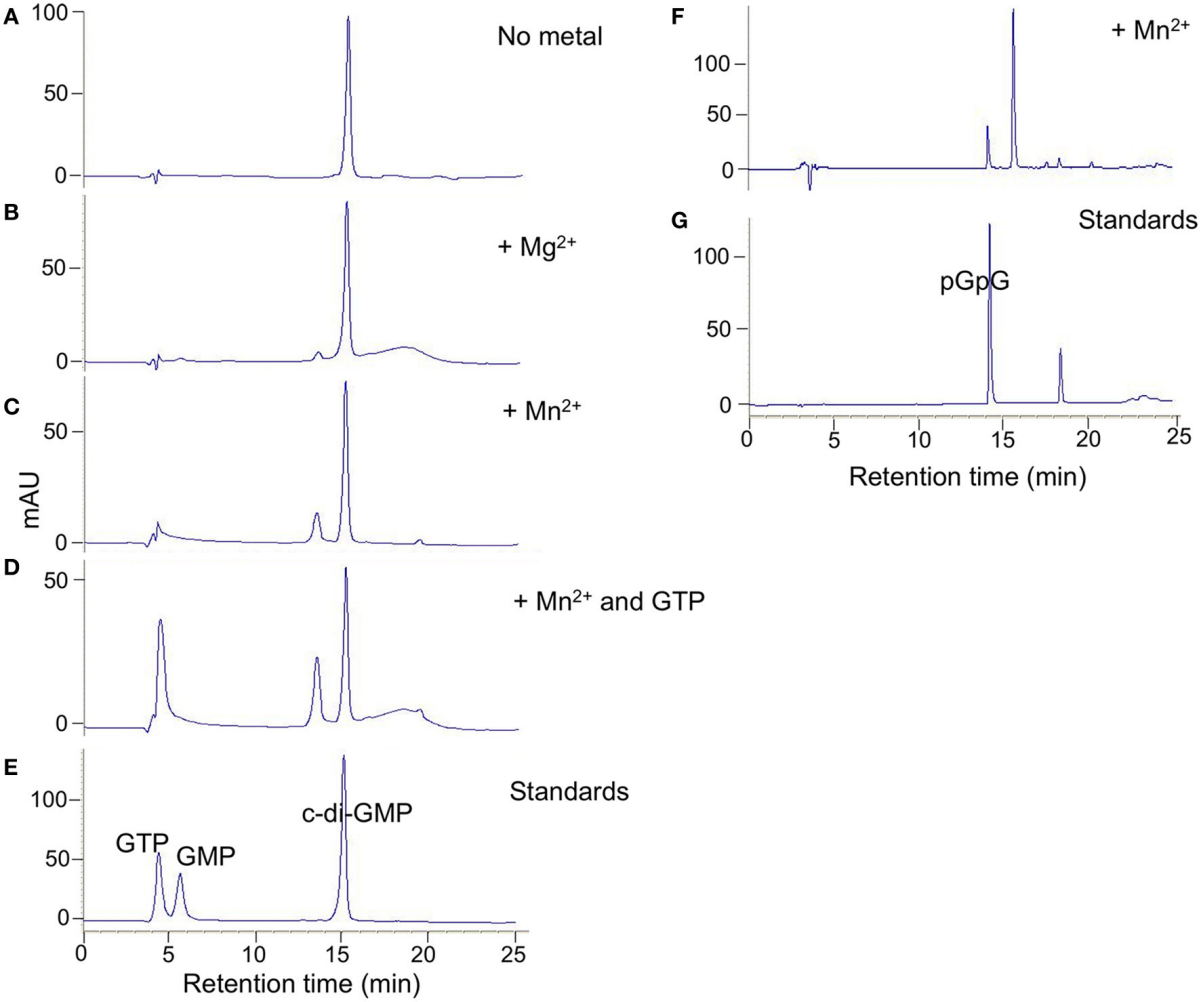
**DVU0408 has PDE activity**. HPLC traces for PDE activity assays with purified DVU0408 and 100 μM c-di-GMP in the presence of no metal **(A)**; 2 mM MgCl_2_ **(B)**; 2 mM MnCl_2_ **(C,F)**; 2 mM MnCl_2_ and 100 μm GTP **(D)**. Standards in **(E)** were a mix of 100 μm GTP, 100 μm GMP, and 100 μm c-di-GMP, while standards run in **(G)** were 100 μm pGpG alone. HPLC traces **(F,G)** were from separate runs than **(A–E)**, and hence the slight shifting of the c-di-GMP and pGpG peaks.

Purified full length DVU0408 protein possessed no detectable DGC activity (not shown). However, it did have PDE activity against c-di-GMP (Figure 4). The amount of product, 5′-pGpG, was greater with Mn^+2^ than with Mg^+2^, whereas no product was formed without a divalent cation. An inactive GGDEF domain is often associated with allosteric activity upon GTP binding (Christen et al., 2005; Kazmierczak et al., 2006); therefore, we tested c-di-GMP hydrolysis activity with Mn^+2^ and GTP. There was an increase in the pGpG peak upon addition of GTP.

#### RRs with HD-GYP domains have PDE activity

**Figure 5:**
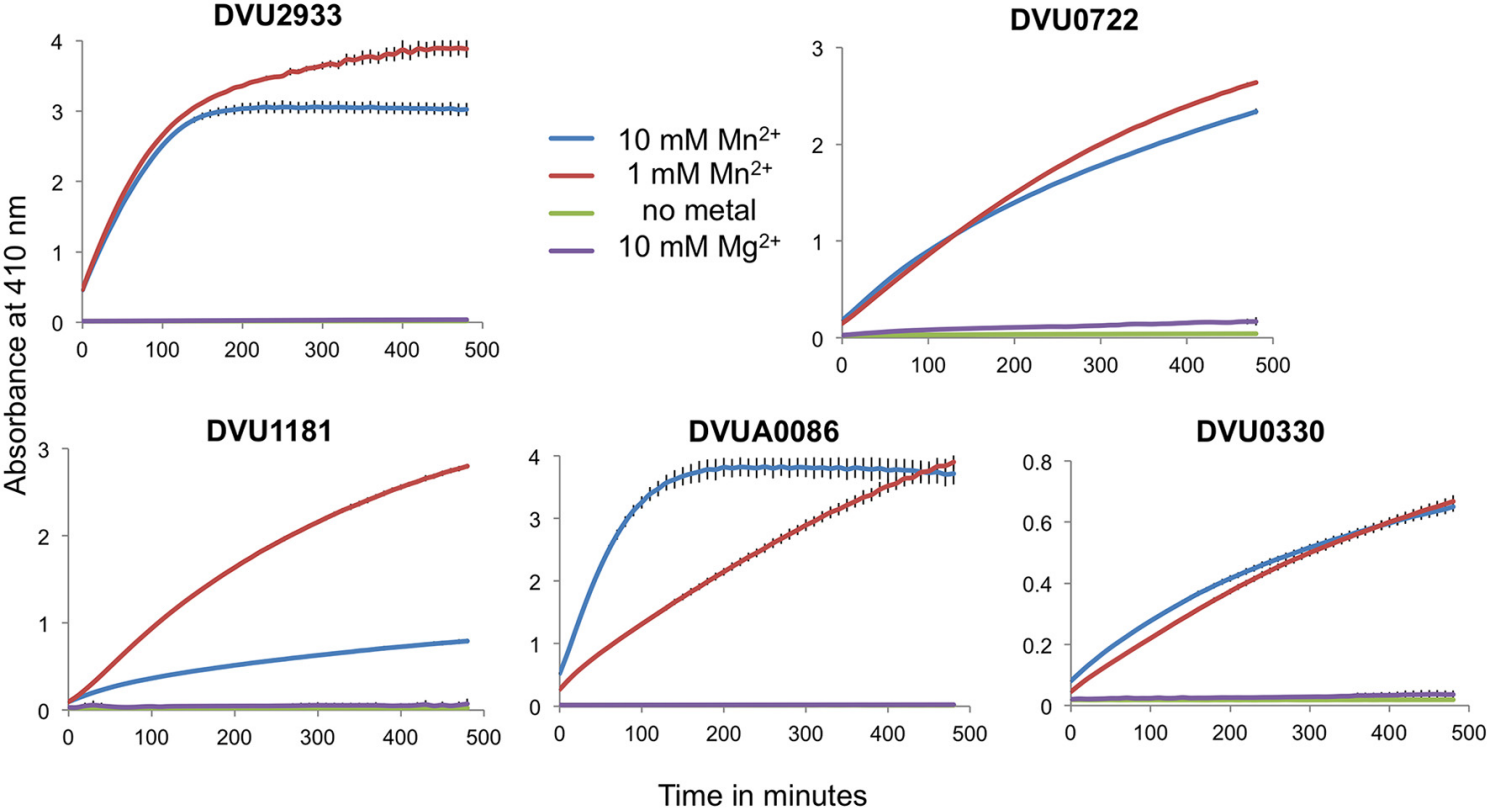
**Isolated HD-GYP domains have PDE activity with bis-*p*NPP**. Isolated HD domains for the RRs were mixed with the synthetic substrate bis-*p*NPP in the presence of either 10 mM or 1 mM MnCl_2_, 10 mM MgCl_2_ or no metal, and the product was measured spectrophotometrically at an absorbance of 410 nm. Data are shown for the mean of triplicate reactions, with error bars indicating standard deviation. Note the differences in the scales on the y-axes.

Full length tagged HD-domain containing RRs were purified and tested for activity against the synthetic substrate bis-*p*NPP as well as against c-di-GMP. The proteins had slow activity against bis-*p*NPP but no activity on c-di-GMP (not shown). For hydrolysis of bis-*p*NPP, the proteins required Mn^+2^ and not Mg^+2^. We attempted to activate the proteins by phosphorylation with acetyl phosphate, however acetyl phosphate reacted with the Mn^+2^ and precipitated. Therefore, we cloned and purified only the respective tagless HD domains. All five purified HD domains showed PDE activity against bis-*p*NPP in the presence of Mn^+2^, but not with Mg^+2^ and not in the absence of any divalent cation (Figure 5). The activity varied among the proteins. DVU2933 and DVUA0086 reached saturation in less than 2 h, DVU0722 and DVU1181 showed slower activity, and DVU0330 that lacks the GYP motif showed very low activity.

**Figure 6:**
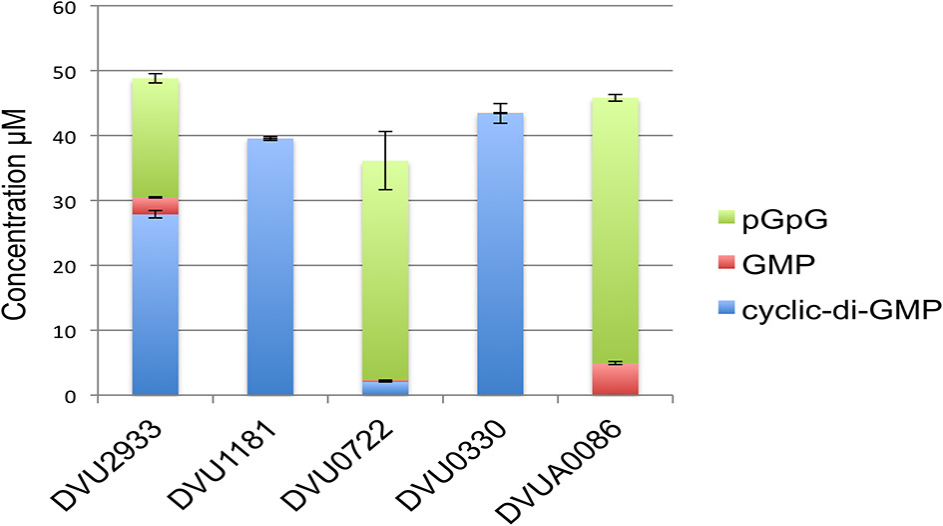
**C-di-GMP hydrolysis activity of HD-domain RRs**. The graph shows the quantitative measurement of the amount of substrate (c-di-GMP) and products (pGpG and GMP) determined by HILIC-TOF MS analysis that were formed after incubation of isolated HD domains of each RR (7–10 μg) with 100 μM c-di-GMP. Concentrations were obtained by comparing peak areas to that of a standard curve. Error bars indicate standard deviations (*n* = 3).

We then tested the purified HD domains for hydrolysis of c-di-GMP (Figure 6). DVUA0086 protein had maximum activity and completely hydrolyzed c-di-GMP, with pGpG as the main product and a small amount of GMP. DVU0722 protein also hydrolyzed most of the c-di-GMP to pGpG. DVU2933 protein had lower activity, but again pGpG was the main product formed. DVU1181 and DVU0330 proteins had no activity against c-di-GMP under the conditions tested.

All five proteins were also tested against other possible substrates such as cyclic-di-AMP, cyclic-AMP, and cyclic-GMP. We did not detect any activity against these substrates (not shown). Providing pGpG as a substrate also showed only as much hydrolysis to GMP as was observed with c-di-GMP as a substrate.

#### Congo red assay

**Figure 7:**
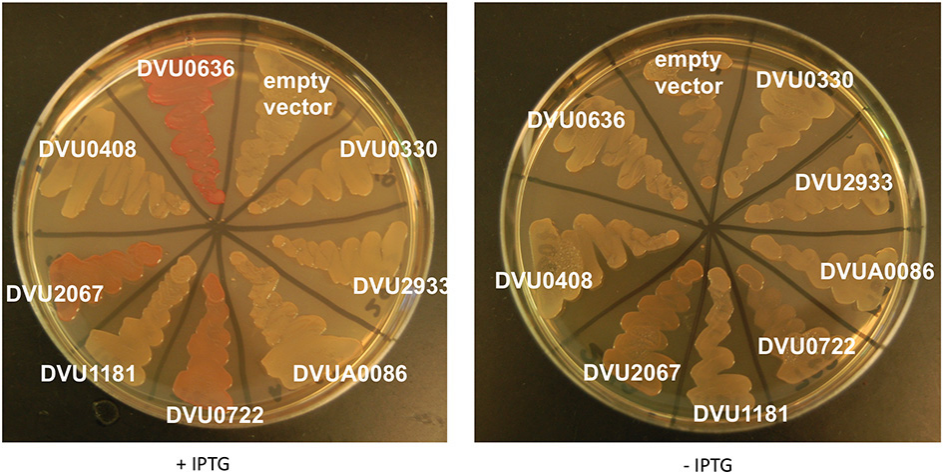
***E. coli* Congo Red binding assay**. *E. coli* BL21 strains expressing full length proteins with C-terminal V5 and His tags were plated on LB-Carb plates with CR (5 μg/ml), with and without IPTG. Plates were incubated at room temperature and imaged after 4 days of growth.

We also tested all eight proteins for their ability to affect cellulose production in *E. coli* with a CR plate assay (Zogaj et al., 2001; Da Re and Ghigo, 2006). A negative result in this assay cannot be used conclusively; a positive response correlates with DGC activity of the expressed gene, and has been reported to evaluate heterologous proteins in *E. coli* (Liu et al., 2010; Ruiz et al., 2011). BL21 strains expressing tagged full length proteins were plated on CR plates ± IPTG (Figure 7). The GGDEF domain containing DVU0636 formed bright pink colonies with CR when induced with IPTG, indicating that DVU0636 had DGC activity *in vivo* as well. The GGDEF domain containing RR DVU2067 formed orange colonies when induced with IPTG, although a slight orange tinge was visible in the absence of IPTG as well. Interestingly, the HD-GYP containing DVU0722 that we showed above to have PDE activity also showed orange colonies on CR plates + IPTG and, similar to DVU2067, a slight coloration was present in the absence of IPTG too.

### Transposon insertion mutant analysis

To further examine physiological roles of these genes, we used mutants with transposon insertions into each of the 8 RR genes that were available from a transposon mutant library.

#### DVU2067 is required for optimal growth on LS4D

**Figure 8:**
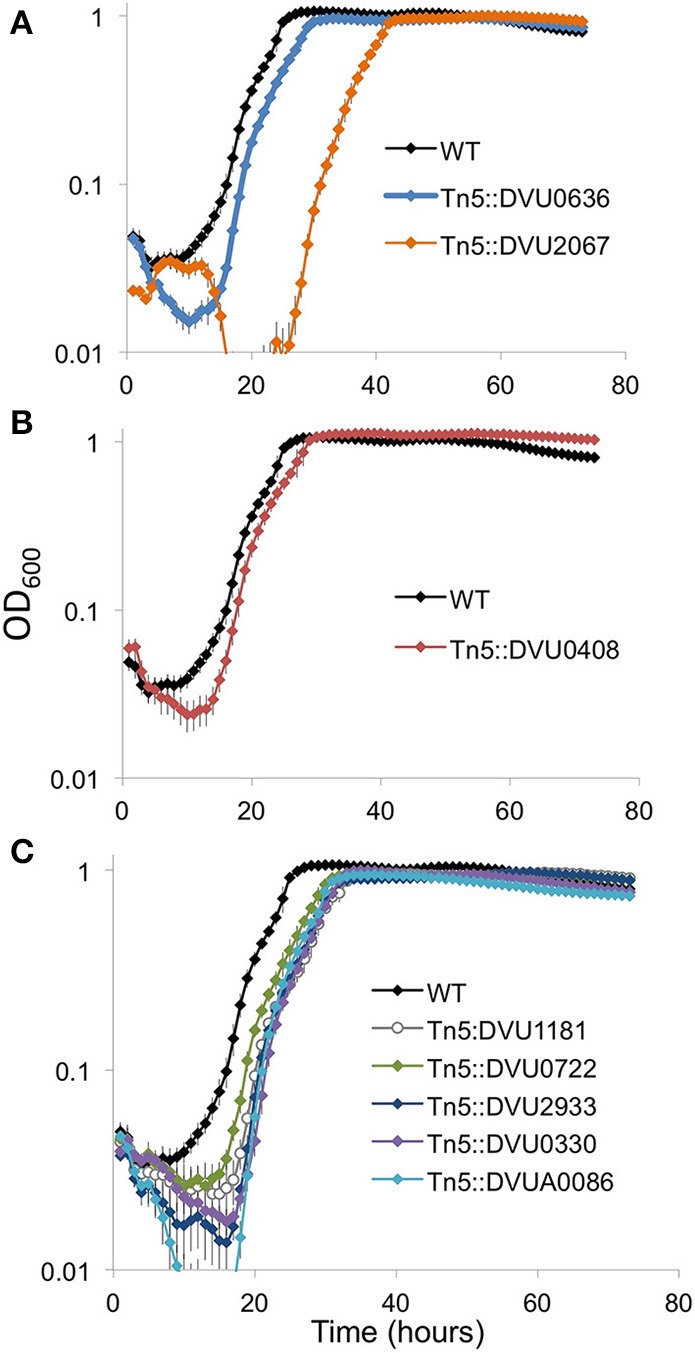
**Growth on LS4D for the transposon insertion mutants**. Transposon mutants in the two GGDEF domain RR genes **(A)**, the GGDEF-EAL RR **(B)**, and the five HD domain RR genes **(C)** were compared to WT during growth on LS4D. Error bars indicate standard deviations (*n* = 4).

We examined the strains for growth defects on LS4D medium. Of the two RRs with DGC activity, the strain with an insertion in DVU2067 showed a prominent growth defect with a longer lag than WT (Figure 8A). Transposon mutant in DVU0636 showed a slightly slower growth and decreased cell mass yields than WT. An insertion mutant in DVU0408 (GGDEF-EAL) showed slightly higher maximal ODs than WT (Figure 8B). All mutants in HD-domain RRs grew similarly with a slightly longer lag and reduced cell densities than WT (Figure 8C).

#### DVU0636 is required for optimal biofilm formation

**Figure 9:**
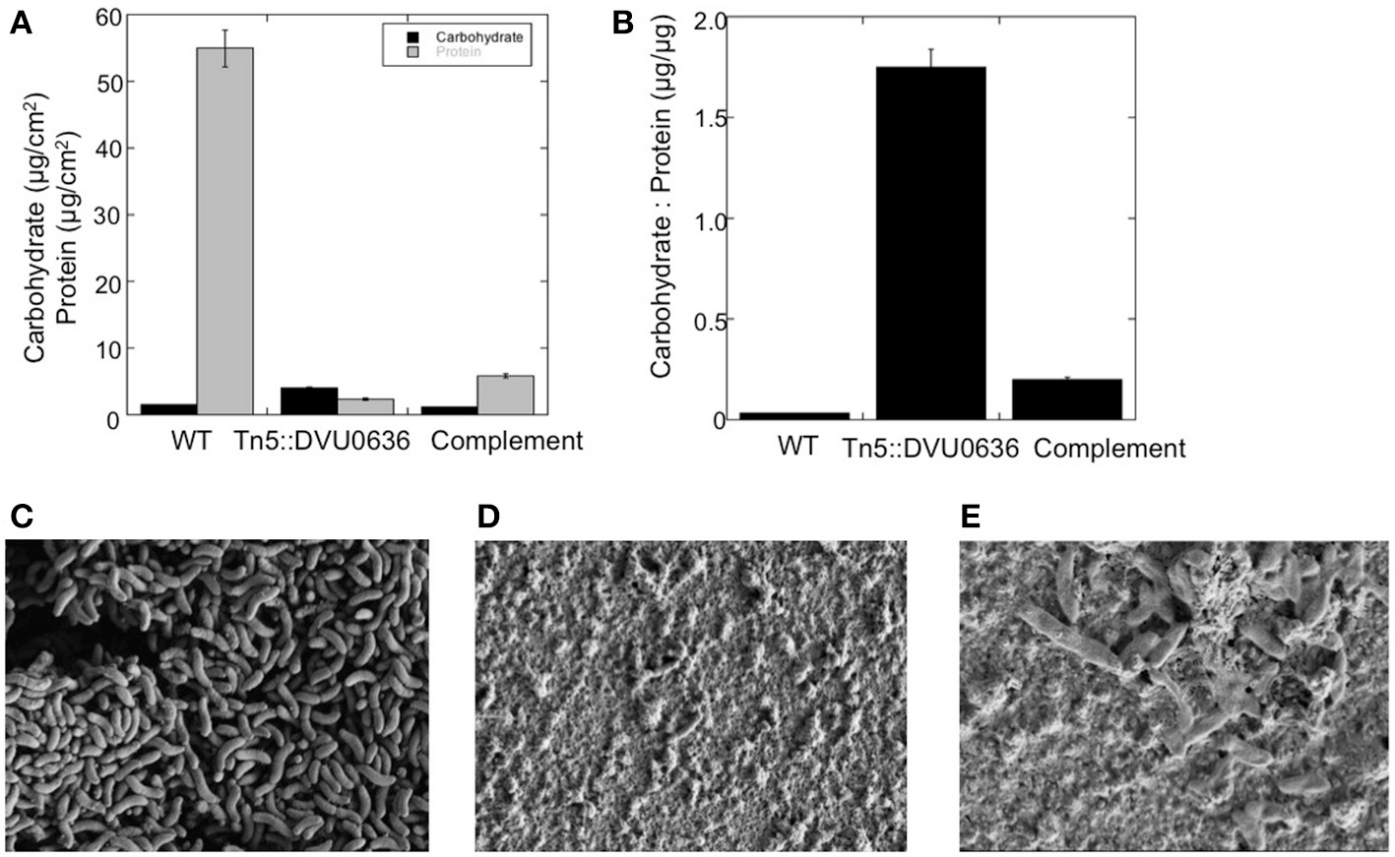
**Inactivation of DVU0636 affects biofilm formation. (A)** The carbohydrate (black) and protein (gray) measurements for WT, Tn5::DVU0636 mutant and complemented strains. Measurements for carbohydrate and protein levels were measured in triplicate for biological duplicates. Error bars indicate standard error of the mean. **(B)** The carbohydrate to protein ratio (μg hexose/μg protein) for wild-type, Tn5::DVU0636 and complemented mutant as steady-state biofilm. **(C–E)** Electron micrographs of biofilms of wild-type at 17,700× **(C)**, mutant at 16,700× **(D)**, and complemented mutant at 22,900× biofilms showing increased cells and decreased extracellular material for WT biofilm compared to mutant biofilm.

Biofilm formation is a common target of c-di-GMP regulation for numerous bacterial groups (Romling et al., 2013), but a relationship between second messenger molecules and biofilm formation has not been investigated previously in δ-*Proteobacteria*. In general, proteins with GGDEF domains that synthesize c-di-GMP promote biofilm formation (Romling et al., 2013). We had strong biochemical evidence for DGC activity with DVU0636 and therefore we examined the transposon mutant in DVU0636 for an effect on biofilm formation. We observed that although the mutant made some biofilm on glass slides, it made less total biofilm and the composition was very different compared to WT biofilm under steady-state conditions (Figure 9). The DVU0636 mutant biofilm had >20-fold lower protein (μg/cm^2^) compared to WT biofilm. Even though there was less biofilm, the mutant biofilm had approximately 2.3-fold more carbohydrate (hexose equivalents, μg/cm^2^) compared to WT biofilm (Figure 9A). *D. vulgaris* WT makes biofilms that are composed more of protein filaments rather than carbohydrates (Clark et al., 2007) and these filaments were not observed in the mutant biofilm. Compared to WT biofilm that has a low carbohydrate to protein ratio (~0.03 μg/μg), the ΔDVU0636 biofilm had an elevated ratio of 1.7 μg/μg (Figure 9B). The FEM images of WT and mutant biofilm also showed biofilm that were drastically different (Figures 9C,D). The WT biofilms contained many more cells that likely accounted for the elevated protein levels, and carbohydrate was not obviously visible. However, the ΔDVU0636 biofilm had fewer visible cells with increased extracellular material that appeared to be carbohydrate. We complemented the mutant strain with a plasmid that expressed DVU0636 under a constitutive promoter. The complemented strain produced a biofilm that was more similar in composition to that of WT with more protein and less carbohydrate (C:P ratio of 0.2 μg/μg) (Figure 9B). Electron micrographs of the complemented mutant biofilm also showed more cells than the mutant (Figure 9E).

## Discussion

The main finding of this study is the identification of a two-component signaling regulator, DVU0636, with significance in biofilm production. Previous work has demonstrated the importance of intracellular interactions and communication for the formation, maintenance, and structuring of biofilms (Burmolle et al., 2014) and this in turn can impact function, stability, and resiliency. However, little is known about anaerobic biofilms that respond differently to mass transport limitations of energy sources other than oxygen. *D. vulgaris* does not produce biofilms with much exopolysaccharide, rather the biofilms are composed of long protein filaments that appear to be flagella or modified flagella (Clark et al., 2007). *In vitro*, we confirmed DVU0636 to be a DGC, where we observed a metal dependence (Mn^+2^ vs. Mg^+2^) on the products formed, though the identity and significance of these peaks remain to be explored. *In vivo*, the overexpression of DVU0636 heterologously in *E. coli* results in a predictable outcome of increased extracellular cellulose production as indicated by increased binding of the CR dye. In *D. vulgaris*, the role of DVU0636 may be in regulating other biofilm related enzymes, suggested by low protein levels in Tn5::DVU0636 biofilms, leading to a much greater C:P ratio relative to the WT or the complemented mutant (Figure 9). Possibly as a result of the non-optimal C:P ratio, the Tn5::DVU0636 biofilm has a “slimier” appearance and is easily disrupted (Figure 9D). There is likely an optimal ratio of protein and carbohydrate for biofilms that is required to maintain biofilm integrity yet maximize transfer of nutrients, and further work is needed to elucidate the environmental cues and regulated systems that bacteria use to coordinate biofilm growth with local environment.

The *D. vulgaris* genome encodes eight two-component RRs with putative c-di-GMP modulating domains. Aside from DVU0636, the other putative cyclase is DVU2067. Of these two RR DGCs, DVU2067 is more highly conserved (Table 2) and is also the enzyme needed for optimal growth typically observed under laboratory cultivation in LS4D medium. None of the other RR transposon mutants presented a significant growth defect in liquid culture. We observed very little *in vitro* activity for DVU2067, and it is possible that this protein requires phosphorylation. The *in vivo* CR plate assay showed that cells expressing DVU2067 gave orange coloration relative to cells expressing DVU0636, which were the more typically reported bright pink. The phenotypes observed for the two RR DGCs suggest that production of c-di-GMP does modulate important functions during both biofilm and planktonic growth.

**Table 2:**
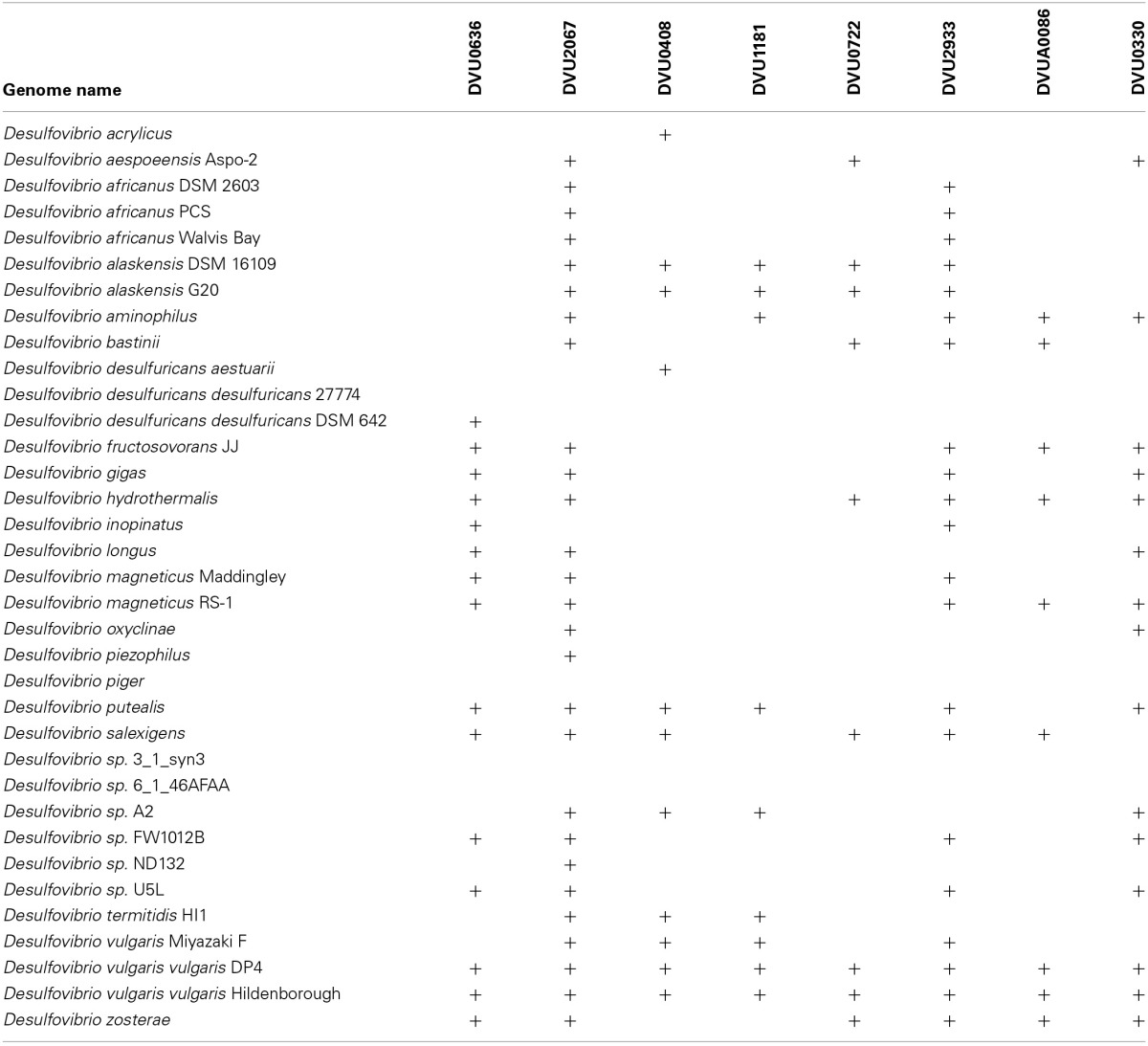
**Conservation of RRs across sequenced *Desulfovibrio***.

Orthologs were determined using the “find homolog” tool on the IMG website (Integrated Microbial Genomes) (Markowitz et al., 2012).

Of the remaining six RRs, we confirmed PDE function for all but DVU0330. DVU0408, which contains both GGDEF and EAL domains, demonstrated turn over of c-di-GMP *in vitro*. The GGDEF domain of DVU0408 has a tyrosine instead of the conserved phenylalanine (Figure 1C), which could explain why this domain is inactive (Malone et al., 2007). However, DVU0408 has an active EAL domain that hydrolyses c-di-GMP into pGpG. Higher activity may be observed in an activated RR. Approximately 33% of GGDEF- or EAL-containing proteins are hybrid proteins with both domains, and, among these hybrid proteins, more than 30% have an active EAL domain combined with an inactive GGDEF domain (Seshasayee et al., 2010). For DVU0408, we observed increased product formation in the presence of GTP, which supports the possibility that the inactive GGDEF domain may bind GTP and provide GTP-dependent control of PDE activity (Christen et al., 2005; Kazmierczak et al., 2006).

With the 4 HD-GYP domain RRs, we observed robust PDE activity with the synthetic bis-pNPP for all but activity with c-di-GMP for only 3. We also observed higher activity with the isolated domains rather than the full-length proteins suggesting that RR activation is necessary for maximal activity. The non-phosphorylated receiver domain of a *Pseudomonas* HD-GYP RR was shown to limit accessibility of the active site to c-di-GMP (Stelitano et al., 2013). The isolated HD-GYP domain for DVU1181 was not active with c-di-GMP although it showed activity against bis-pNPP. Perhaps a more active conformation involving the receiver domain is required for activity with c-di-GMP. The HDOD domain, as present in DVU0330, is related to the HD superfamily of phosphohydrolases (Aravind and Koonin, 1998) but lacks the key active site residues (Galperin, 2006). An HDOD RR, GsmR, was recently characterized from *Xanthomonas campestris*, and found to regulate the expression of motility related genes. GsmR lacked the conserved His in the active site, and also lacked phosphatase or phosphodiesterase activity (Liu et al., 2013). DVU0330 has the conserved HD active site residues (Figure 1C), and here we showed that the isolated domain has weak PDE activity with bis-*p*NPP and none with c-di-GMP or any of the other substrates.

We also observed that for DVU0408, as well as the three active HD-GYP RRs, the hydrolysis product was primarily pGpG. For EAL domains it has been reported that pGpG is the primary product, however GMP is reported as the predominant product for HD-GYP domains (Ryan et al., 2006; Romling et al., 2013). Some recent studies on other HD-GYP proteins also report on pGpG being the main product (Stelitano et al., 2013), and they suggest the possibility of pGpG being a signaling molecule in its own right. *D. vulgaris* Hildenborough is among the genomes with the highest number of HD-GYP domain genes encoded (Galperin et al., 2010) with a total of 14 genes, and it would be interesting to know which perform the incomplete hydrolysis of c-di-GMP to pGpG vs. completely to GMP.

As expected, the CR plate assay with *E. coli* strains expressing PDE RRs gave white colonies with or without IPTG. The interesting exception was the strain expressing DVU0722, which had orange coloration. This was unexpected as DVU0722 has PDE activity *in vitro* and hydrolyzes c-di-GMP. We speculate that DVU0722 interacts with other proteins in *E. coli* and ultimately leads to increased cellulose production.

Gene knockout strains of PDE RRs resulted in no impact on growth in liquid cultures. Additional experimentation is required to identify the phenotypes that involve the other RRs. Genetic data are available for only a few HD-GYP proteins from pathogens, and none from environmental isolates. RpfG from *Xanthomonas campestris* promotes synthesis of virulence factors and affects biofilm formation and motility (Ryan et al., 2010, 2012). The *Borrelia burgdorferi* PdeB controls motility and promotes virulence in ticks (Sultan et al., 2011). Two HD-GYP domain proteins from *Pseudomonas aeruginosa* control swarming motility and production of virulence factors (Ryan et al., 2009). However, other studies conducted in *D. vulgaris* Hildenborough do shed some light on potential conditions under which the function of these RRs may be relevant. For example, in a recent evaluation of all transcriptionally acting two-component RRs in *D. vulgaris* (Rajeev et al., 2011), DVU0408 was found to be the target for the RR DVU1063, which also targets several flagellar genes. DVU0408 may thus be involved indirectly in cellular motility. A knockout of this gene however had motility in wet mounts as well as in soft agar plates (data not shown). In the same study (Rajeev et al., 2011), DVU2933 was found to be part of a five-gene operon that also encodes a histidine kinase and another RR, DVU2934, which is a DNA binding RR that targets the *lpxC* gene involved in lipid A synthesis.

**Figure 10:**
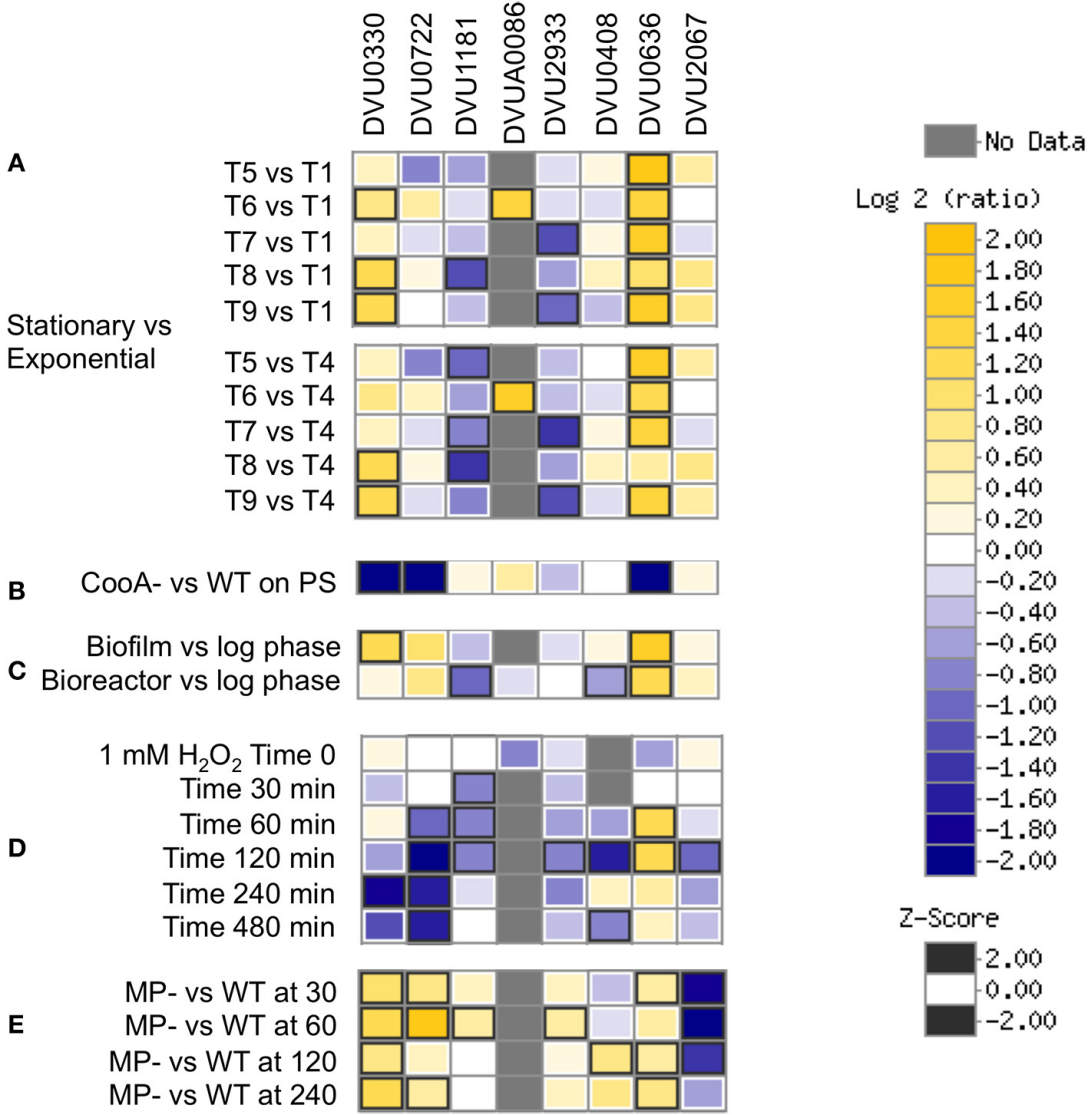
**Transcriptomics data showing relevant conditions for differential expression of the 8 RRs. (A)** Stationary vs. Exponential phase where T1-T2 are exponential phases, T3-T4 are late exponential phases, T5 is early stationary, and T9 is late stationary (Clark et al., 2006). **(B)** CooA mutant (Deletion in DVU2097) vs. WT on pyruvate sulfate (Rajeev et al., 2012). **(C)** Biofilm and bioreactor samples vs. log phase cells (Clark et al., 2012). **(D)** Temporal analysis of exposure to 1 mM hydrogen peroxide (Zhou et al., 2010). **(E)** pDV1- strain vs. WT (unpublished). Data were visualized using MicrobesOnline (Dehal et al., 2010).

A review of available transcriptomics measurements in *D. vulgaris* (Figure 10) shows increased expression of DVU0636 (and DVU0330) transcripts in stationary phase vs. exponential growth stages (Clark et al., 2006). DVU0636 was also upregulated during biofilm growth (Clark et al., 2012), which is again consistent with our results. DVU0636 was also increased in expression during peroxide exposure, in contrast to DVU1181, DVU0722, and DVU2933, which were decreased in expression in this condition (Zhou et al., 2010). In a more environmentally relevant growth regime, defense against peroxide and other oxidative stresses may include the production of biofilms and may explain the increase in DVU0636 transcripts. Further, DVU0636 (and DVU0330, DVU0722) were decreased in expression, while DVU2067 was increased in expression in a CooA mutant, which lacks the gene DVU2097 that acts as a carbon monoxide sensor. Transcriptomic analysis of the CooA mutant showed changes similar to that seen in WT when exposed to CO including a decrease in expression of Fur regulon genes, and was suggested to have been due to increased levels of endogenous CO and oxidative stress (Rajeev et al., 2012). Conversely, DVU0636 is upregulated, while DVU2067 is downregulated in the pDV1-minus strain, relative to the wild type. The pDV1 minus strain lacks the native 200-kb plasmid present in wild type *D. vulgaris* Hildenborough and has a reported defect in attachment and biofilm formation (Clark et al., 2007) and is also missing the DVUA0086 RR. More experimentation is required to prove if the regulation and function of these RRs are interconnected or interdependent.

## Conclusions

This is the first demonstration of PDE and DGC activity in a sulfate-reducing bacterium. We were most successful in identifying conditions under which the two DGC RRs are important, though they may have a role in other conditions also. The most conserved candidate, DVU2067, generated a growth defective strain in planktonic cultures. The less conserved, DVU0636, had no impact during growth in liquid cultures but had a clear role in biofilm production. The impact on biofilm growth manifested in altered macromolecular composition that caused decreased biofilm maintenance with elevated carbohydrate levels. The PDE RRs have no clear roles in WT planktonic growth, though some ancillary data links the dual domain DVU0408 with flagellar genes and PDEs to stress responses. Further studies will reveal the roles they play in other aspects of *D. vulgaris* physiology.

### Conflict of interest statement

The authors declare that the research was conducted in the absence of any commercial or financial relationships that could be construed as a potential conflict of interest.
